# Free flight odor tracking in Drosophila: Effect of wing chemosensors, sex and pheromonal gene regulation

**DOI:** 10.1038/srep40221

**Published:** 2017-01-09

**Authors:** Benjamin Houot, Vincent Gigot, Alain Robichon, Jean-François Ferveur

**Affiliations:** 1 Centre des Sciences du Goût et de l’Alimentation, UMR6265 CNRS, UMR1324 INRA, Université de Bourgogne Franche-Comté, 6, Bd Gabriel, 21000 Dijon, France; 2 UMR INRA/CNRS/UNS 7254, Institut Sophia Agrobiotech, 400 route des Chappes, P.O. Box 167, 06903 Sophia Antipolis, France

## Abstract

The evolution of powered flight in insects had major consequences for global biodiversity and involved the acquisition of adaptive processes allowing individuals to disperse to new ecological niches. Flies use both vision and olfactory input from their antennae to guide their flight; chemosensors on fly wings have been described, but their function remains mysterious. We studied Drosophila flight in a wind tunnel. By genetically manipulating wing chemosensors, we show that these structures play an essential role in flight performance with a sex-specific effect. Pheromonal systems are also involved in Drosophila flight guidance: transgenic expression of the pheromone production and detection gene, *desat1*, produced low, rapid flight that was absent in control flies. Our study suggests that the sex-specific modulation of free-flight odor tracking depends on gene expression in various fly tissues including wings and pheromonal-related tissues.

400 millions years ago, when insects gained the ability to fly, they started to conquest the space overhanging most terrestrial environments^1^. Flying ability has likely spread in most insect orders likely because it provides many advantageous functions. Flight allows insects to escape from ground predators, to predate other animals^2^, to explore new ecological niches inaccessible to terrestrial animals^3^, and to migrate—sometimes over very long distances—in the search of sex partners and new food sources, this increasing their dispersion^4^,^5^,^6^,^7^.

To lift up from the ground and navigate in space, insects need to constantly integrate the feedback of multiple sensory modalities provided by visual, chemical and mechanosensory signals^8^. Orientation to sensory cues, and specially to the chemical signals provided by food and conspecifics, requires a very sophisticated ability. Indeed, during flight, insects face atmospheric turbulence which strongly impact on the dispersion of molecular cues and vortex gradients of odorants, rendering it difficult to trace the emitting source^9^. The 240 000 known species of Diptera possess a single pair of wings while the second pair—generally present in most other insect orders—is transformed in halteres acting as gyroscopes helping them to keep a balance during flight^10^,^11^.

During the last two decades, the study of the dipteran model species *Drosophila melanogaster* has expanded our knowledge on some of the fundamental mechanisms underlying tethered and powered insect flight. In particular, the use of genetic mutants targeting a limited number of cells has permitted to identify some of the muscular and neural components involved in the physical ability to fly^12^,^13^,^14^. For example, three dopaminergic neurons of the ventral ganglion are involved in the bilateral coordination of wings during tethered flight^15^, whereas octopaminergic neurons projecting in the visual lobes modulate vision-dependant flight^16^,^17^. Varied sensory cues can influence Drosophila flight: olfactory stimuli provided by food and carbon dioxide strongly attract flies during flight^18^,^19^ as do visual cues related to motion and to polarized light^20^,^21^. During flight, the effect of visual cues can be modulated by mechanosensory stimuli^22^,^23^ and also by olfactory cues which are bilaterally perceived by flies^24^,^25^,^26^. Recently, chemosensory organs on the wing anterior margin, were shown to be involved in gustatory perception^27^.

The effect of temperature, age and experience has been estimated in the flight performance of Drosophila and in other insects^28^,^29^, contrary to sex-specific variation which was rarely investigated^30^. Here, we used a wind tunnel to compare free flight performance in mature male and female flies of transgenic and wild-type strains in response to varied odorant cues and principally to laboratory plain food. Using *D. melanogaster* transgenics, we manipulated chemosensory hairs on the wing anterior margin^27^. We also measured the effect of *desat1*, a gene involved in several aspects of pheromonal communication, and expressed both in neural and non-neural tissues^31^,^32^.

## Results

### Flight performance to varied odorant stimuli in a control *D. melanogaster* strain

**Figure 1:**
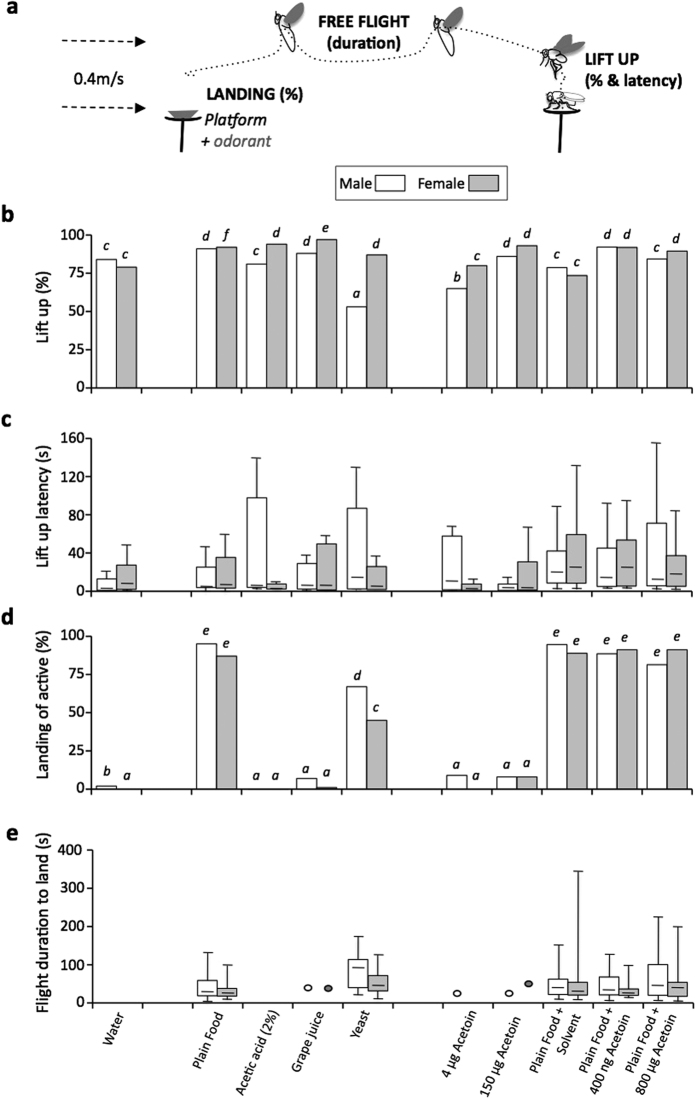
Free-flight of *D. melanogaster* flies of the Cs strain to various odorant stimuli. (**a**) Schematic representation of free-flight in the wind tunnel. The four flight features measured are: lift up frequency, lift up latency, flight duration and landing frequency; The wind speed is indicated on the left. Histograms and box- whisker plots below (**b**–**e**) represents these four parameters of free-flight in mature male and female flies (empty and shaded bars, respectively). “Frequency data” are shown as histogram bars whereas “duration data” are shown as box-whisker plots. Each bar of the box-plot represents the 50% median data (or second and third quartile; the median value is shown as a small horizontal bar) while the two thin vertical bars (“whiskers”) down and up the box represent the 25% lower and 25% higher data distribution (fourth and first quartiles), respectively. (**b**) The frequency of lifting-up, (**c**) the lift-up latency and (**d**) among flying insects, the proportion of flies landing on the platform with water and (**e**) their flight duration between lift-up and landing. Free-flight was measured toward water, plain laboratory food, acetic acid, grape juice, yeast, acetoin alone or mixed with plain food) at two concentrations, and the solvent used for acetoin. The difference between lift-up and landing frequencies were tested with a Fisher exact test, whereas time durations (for the lift-up latency and flight duration) were assayed with a Kruskall-Wallis test. For each parameter, significant differences are indicated by different letters at level p = 0.05. N = 23–38, except for males and females to water (61–65) and for males to 150 μg acetoin (14).

First, free flight to several odorant stimuli was measured in mature *D. melanogaster* male and female flies of the Canton-S (Cs), a standard laboratory strain (Fig. 1). We compared four flight features: lift-up frequency and latency, landing frequency (among flying insects) and flight duration until landing (Fig. 1a). We tested the effect of water (used as a “empty control” substance), plain laboratory food, grape juice and acetic acid (two potentially attractive substances^33^,^34^) and yeast (an active component of plain food). We also measured the effect of acetoin, a volatile molecule—alone or mixed at two concentrations with plain food—produced both by fermenting fruits and by Drosophila larvae^35^,^36^.

Flies generally showed a high lift-up frequency (70–95%; except for males with yeast = 50%; Fig. 1b). With most odorant sources, but not with control odors, females lifted up more frequently than males (Fisher exact test; p = 0.0033). Lift-up latency was not significantly affected by the nature of the stimulus nor by the sex: it generally occurred around 40–60 sec (Fig. 1c; K_19d.f_ = 75.3; p = ns). However, some of the males tested with acetic acid, yeast and 4 ug acetoin showed a very delayed lift-up latency compared to most flies of other genotypes. Landing frequency varied according to odorant sources, but rarely diverged between the sexes. Only plain food (alone or mixed with acetoin and/or its solvent)—and to a lesser extent yeast—induced relatively high landing frequencies on the “odorant” platform (Fig. 1d; for plain food: 85–95%; for yeast: 45–75% with a higher landing frequency in males than females; K_19d.f_ = 39.94; p = 0.003). As measured from lift-up to landing, flight generally lasted 30–90 sec, with no obvious stimuli- or sex-related effect (Fig. 1e).

### Role of wing chemosensory organs in flight performance

**Figure 2:**
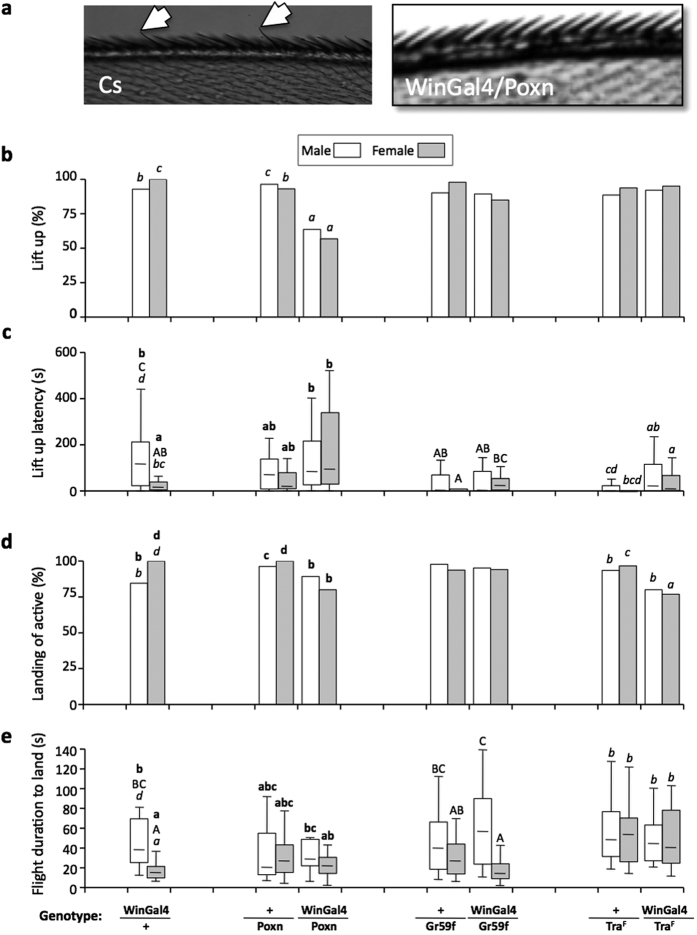
Free-flight of transgenic Drosophila with genetically altered wing margin chemosensors to plain food. (**a**) Pictures of the wing anterior margin in wild-type (Cs, left; arrows indicate chemosensory hairs) and WinGal4/Poxn transgenic flies (right; note the absence of chemosensory hairs). (**b**–**e**) From top to bottom, histograms and box plots represent the four free-flight features (described in Fig. 1a) to plain food. The same genotypes were tested toward water (see Supplementary Fig. S1). The simplified genotypes are indicated below each pair of bars (males/females). Flies carry the following transgenes: WinGal4 is the driver transgene expressing Gal4 in the wing anterior margin sensory organs; Poxn is the UAS-*poxneuro*-RNAi reporter transgene allowing to transform chemosensory sensilla into mechanosensory sensilla in Gal4-targeted tissues; Gr59f is the UAS-RNAi of the Gr59f gustatory receptor. Tra^F^ is the UAS-tra^F^ transgene used to feminize Gal4-targeted tissues. For exemple, flies of the “WinGal4/Poxn” genotype simultaneously carry the WinGal4 and the Poxn transgenes leading to the transformation of wing chemosensory into mechanosensory sensillae. “+” corresponds to a wild-type chromosome. In this figure, we statistically compared genetically-related genotypes. The WinGal4 genotype was used in the comparison involving three data series: “Poxn”, “Gr59f “and “Tra^F^”. Each series of statistical tests is represented by different font letters (bold lower case, capital, italic, respectively). For other statistical details, please refer to the Fig. 1 legend and (see Supplementary Table S1). N = 28–51.

We manipulated the chemosensory hairs located on the anterior wing margin to investigate their possible effect on odor-tracking flight. The WinGal4 driver transgene, showing restricted expression to these organs^27^, was used to target several UAS-reporter transgenes (Fig. 2; see Materials and methods). The genetic transformation of wing chemosensory sensilla into mechanosensory sensilla with the UAS-*poxn*-RNAi transgene produced WinGal4/Poxn flies (Fig. 2a) that lifted-up less frequently than their transgenic controls (WinGal4/+ and +/Poxn) both to plain food and water (Fisher exact test; *P* < 0.001; Fig. 2b; Supplementary Fig. S1 and Tables S1 and S2). WinGal4/Poxn flies showed no other obvious flight alteration. Differently, with plain food transgenic control WinGal4/+ females lifted-up and flew faster than sibling males (Fig. 2c,e; Supplementary Table S1; KW_5df_ = 21.6 and 19.7, respectively; *P* < 0.001) whereas no such sexual difference was detected in WinGal4/Poxn flies. This indicates that Gal4 expression in targeted wing sensilla induced this effect which was abolished after the genetic transformation of these tissues. To further test the sex-specific effect of WinGal4 on both flight parameters, we feminized wing-margin sensory organs with the UAS-*tra*^F^ reporter transgene (Tra^F^). Indeed, WinGal4/Tra^F^ flies showed no sexual difference for neither parameter supporting the existence of a functional sexual dimorphism in the wing-targeted chemosensory sensilla.

However, targeting the RNAi of the Gr59f gustatory receptor in wing margin sensilla (of WinGal4/Gr59f flies) only abolished the sexual difference observed for lift-up latency (p = ns) but not for flight duration (KW_5df_ = 55.4; *P* < 0.0001; Supplementary Table S1) indicating that Gr59f maybe involved in sexually dimorphic factors underlying lift-up latency.

### Role of *desat1* in flight performance

**Figure 3:**
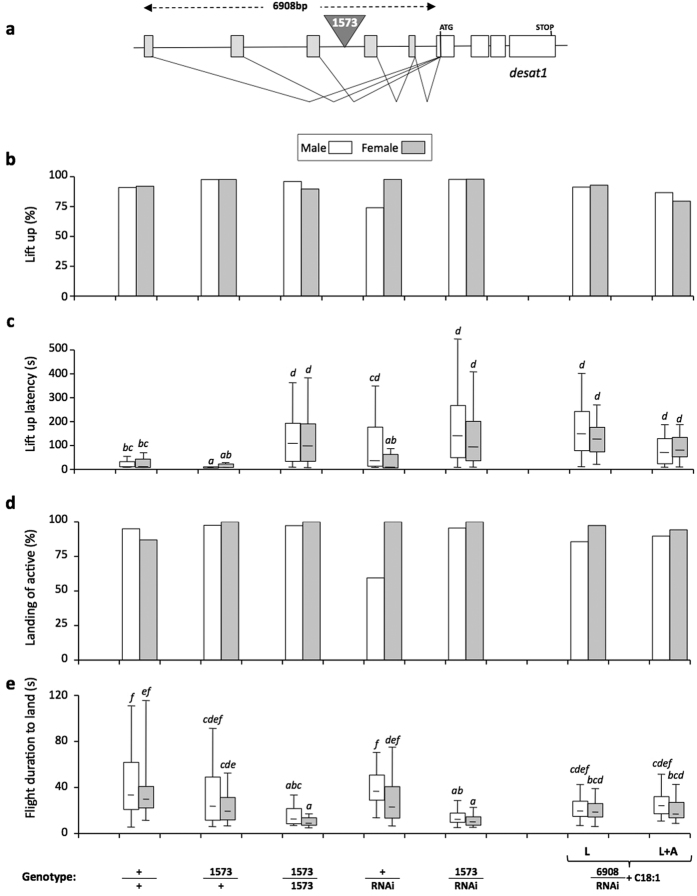
Free-flight to plain food in *desat1* transgenics. (**a**) Schematic representation of the molecular structure of the *desat1* pheromonal gene. The inverted triangle (1573) indicates the PGal4 element inserted in *desat1* acting both as a mutation (in homozygous 1573/1573 flies) and as a Gal4 driver used to target the UAS-*desat1*-RNAi transgene (1573/RNAi). The complete *desat1* regulatory region (6908 bp) was also used to target UAS-*desat1*-RNAi. (**b**–**e**) The four histograms series represent four free-flight features (see Fig. 1a) toward plain food (for the response to water, see Supplementary Fig. S2). The simplified genotypes are indicated below each pair of bars. Beside the wild-type genotype (+/+), flies either contained one or two copies of the 1573 inserted element (1573/+, 1573/1573, respectively). We also targeted the UAS-*desat1*-RNAi transgene with a single copy of the 1573 allele (1573/RNAi), or of the complete *desat1* regulatory region fused with Gal4 (6908-Gal4). In the latter genotype (6908/RNAi), adult rescue was obtained with C18:1-rich diet provided only during larval stage (L) or during both larval and adult stages (L + A). Given their intricated relationship, the statistical difference of all genotypes was simultaneously tested. For details, please refer to Fig. 1 legend and Supplementary Table S3. N = 40–65.

Next, we assessed the effect of *desat1*, a gene involved in multiple aspects of pheromonal communication, on the flight of *desat1* transgenics (Fig. 3). First, 1573/1573 flies (homozygous for a P-Gal4 element inserted in the *desat1* regulatory region^37^; Fig. 3a), showed an increased lift-up latency to plain food (compared to “+” and “1573/+” control genotypes; KW_15d.f._ = 217.8; *P* < 0.0001; Fig. 3b; Supplementary Table S3a), and to water (Supplementary Fig. S2 and Table S4). Moreover, 1573/1573 mutant females flew much faster to plain food (median value = 8 sec) than control genotypes (29 and 19 sec, respectively; K_215d.f._ = 183.7; *P* < 0.0001; Fig. 3e and Supplementary Table S3b). In males, the flight duration of 1573/+ was intermediate between that of + and 1573/1573 mutant flies. A similar delayed lift-up and faster flight alteration was found in flies with a single 1573 allele copy (1573-Gal4) targeting the UAS-*desat1*-RNAi transgene (1573/RNAi).

We also tested the flight of 6908/RNAi flies with the whole *desat1* regulatory region (6908bp-Gal4^32^,^38^) targeting UAS-*desat1*-RNAi (6908/RNAi). To yield adults, 6908/RNAi larvae were raised on plain food enriched with oleic acid (“L”)^39^. Some 6908/RNAi individuals also received this diet during adult life (“L + A”). 6908/RNAi flies (L and L + A) only showed a slightly delayed lift-up latency without any other obvious defect.

**Figure 4:**
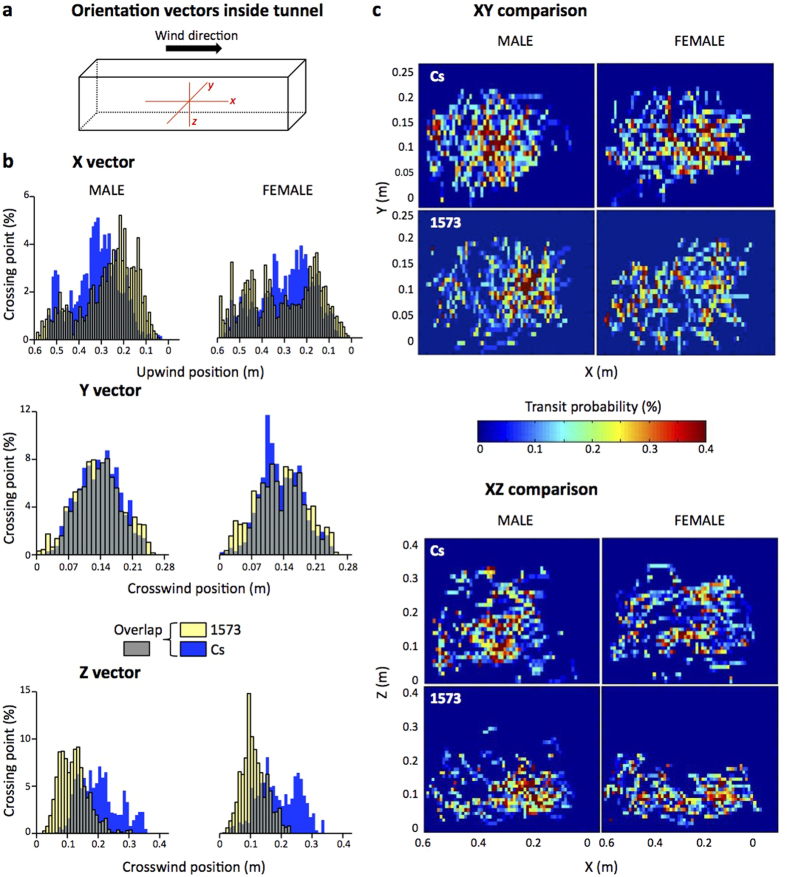
Three-dimensional distribution of flies in the wind tunnel during free-flight to plain food. (**a**) During flight, the distribution of flies was computed according to each axis of the wind tunnel: X = length, Y = width, Z = height. (**b**) For each sex (male = left; female = right) and genotype (Cs control flies = blue; 1573 mutant = yellow; overlap = grey), histograms represent the unidimensional probability to find a fly on each axis (vector). On X axis, a reduced upwind position (close to “0”) indicates the proximity to the point of release (and lift-up). For the Y and Z axis, the medium point of the axis (0.14 and 0.2 m) represents the middle width and height into the tunnel, respectively. (**c**) The two-dimensional heat map representations reflect the probability to find a fly in the tunnel according to XY (top) and XZ (bottom) 2D-plots. The responses of males and females (left and right, respectively) of the control and mutant genotypes (top and bottom, respectively) were compared to plain food. N = 23–41 (For the response to water, see Supplementary Fig. S3).

To explore more precisely the altered flight of 1573/1573 mutant flies (hereafter named “1573”), we measured their probability to be detected in the three tunnel axes (X = length; Y = width; Z = height; with plain food or water: Figure 4a and Supplementary Fig. S3, respectively). With plain food, both 1573 and Cs flies diverged for their distribution on two X and Z axes (Fig. 4b). On the X axis, 1573 males were more frequently found at a closer distance of the lift-up platform compared to Cs males whereas Cs females showed a more central distribution peak than 1573 females. On the Z axis, both mutant males and females were clearly distributed at a lower height (0–0.2 m) than Cs flies. These differences can be visualized on XZ bidimensional “heat-map” plots corresponding to the side-view of the tunnel (Fig. 4c). With water, the “mutant *vs*. Cs” difference was less marked (Supplementary Fig. S3) except on the X axis where mutant flies (compared to Cs) showed a similarly biased distribution as with plain food (Fig. 4). To describe more exhaustively the variant flight of 1573 flies, we also measured the total distance travelled, velocity, heading flight, and angular velocity (Supplementary Fig. S4). With water, but not with plain food, 1573 flies landed after a shorter flight distance (Supplementary Fig. S4a). The velocity of mutant females was higher as compared to (*i*) mutant males (for both stimuli), and to (*ii*) control flies (only to plain food; Supplementary Fig. S4b; *F* = 13.86; *P* = 0.002). Heading flight was generally negative or close to “0”, except in mutant females to plain food where it was positive (Supplementary Fig. S4c; *F* = 46.38; *P* < 0.0001). This difference is also visualized on polar histograms (Supplementary Fig. S5). The angular velocity (of both sexes to plain food and water) was higher in 1573 flies than in Cs flies (Supplementary Fig. S4d; *F* = 15.34; *P* = 0.0001) indicating that mutant flies showed more turning events than control flies during their shorter-duration flight.

## Discussion

Our data obtained with *D. melanogaster* transgenics suggest that wing chemosensors and *desat1*-expressing tissues affect precise features of odor-tracking free-flight. The chemosensory sensilla harbored by the Drosophila wing anterior margin house enzymatic, ionotropic and receptor proteins potentially involved in chemosensation (odorant binding proteins, cytochrome P450, Pickpoket 25^27^,^40^,^41^,^42^,^43^,^44^). Some wing chemosensors respond to tastant molecules (glucose, trehalose, quinine, denatonium^27^), and can change chemo-induced aggregation behavior. However, their role in olfactory-driven free flight behavior remained unexplored. Our experiments suggest that wings chemosensors can modulate the decision to lift-up and, in a sex specific manner, the speed of odor-guided flight.

The genetic transformation of wing chemosensors decreased lift-up frequency in male and female flies to both plain food and water (Fig. 2b,c and Supplementary Fig. S1a) whereas WinGal4/+ transgenic females lifted-up faster (to plain food and water) and flew faster (to plain food) than their males (Fig. 2d,f and Supplementary Fig. S1d). This sexual difference was abolished in males with genetically feminized or ablated wing chemosensors. This indicates that some of the wing sensors targeted by WinGal4 sex-specifically influenced flight duration. The defect induced by a single copy of the WinGal4 transgene may result of Gal4 toxicity in the chemosensory nervous system^45^,^46^,^47^. To our knowledge, no sex-specific difference for the wing margin chemosensors, or for their projection in the central nervous system, or for flight behavior has been reported in Drosophila^44^. This maybe due to the fact that most—but not all—Drosophila flight-related studies only dealt with one sex^30^. The sex-specific effects reported in two most relevant studies rather reflect a sexual difference in walking—but not flying^48^—, or may be due—not only to wings but—to all sexually dimorphic chemosensory tissues of the adult^34^,^40^,^49^,^50^.

The manipulation of the pheromonal system also affected several aspects of free flight odor tracking. If the delayed lift-up latency shown by both 1573/1573 and 1573/RNAi—and to a lesser extent by 6908/RNAi—flies could be explained by RNAi targeting cells involved in fat supply (fat body; oenocytes), this preventing the fly to engage in a high energy-consuming behavior (as in other insects^51^), the faster flight to plain food of 1573/1573 and 1573/RNAi flies remains unexplained. This effect (not linked to the distance travelled, the velocity or the heading flight which were similar in mutant and control flies; Supplementary Fig. S4a–c), was likely due to the increased angular velocity shown by mutant flies. This indicates that mutant flies changed direction more often when flying upwind (along the X axis) than control flies which showed a straighter flight. Control flies can modulate their flying pattern according both to the distance and quality of the odorant stimulus: at far, their fast upwind flight keeps a constant heading, but when they get closer to a stimulatory odor, their heading flight decreases more than with clean air^18^. During this approach, flies modulate the directionality of their flight by changing both wingbeat frequency and amplitude^25^,^26^,^52^,^53^.

Mutant 1573 flies showed a faster flight with a higher angular velocity which was also performed at a much lower altitude than in control flies. This “lower and faster zig-zag” flight pattern could reflect a skewed alteration of olfactory perception in the mutant resulting of a spatially unbalanced *desat1* misexpression in chemosensory tissues normally expressing this gene^31^,^32^. This hypothesis is supported by the mildly altered flight shown by ubiquitously targeted 6908/RNAi flies as compared to the strong changes observed in 1573 mutant flies. This indicates that the simultaneous alteration of all chemosensory *desat1*-expressing organs did not induce a spatially unbalanced chemosensory perception differently to the *desat1*-1573 mutation which only affected part of the chemosensory system^32^,^37^. Moreover, the larval fat-rich diet may have cured some of the 6908-Gal4 genetically induced defects.

Apart the Gr59f gustatory receptor protein which may be involved in the lift-up latency response, we currently do not know which other chemosensory receptors involved in the free flight response to food. We believe that other gustatory, ionotropic and Pickpocket wing margin receptors are also involved in this complex olfactory-driven behavior given that such receptor proteins can fulfill diverse non-gustatory functions and modulate the flight response to volatile stimuli^27^,^54^,^55^.

In nature, the 1573 mutant flight may be maladaptive if the time spent exploring the immediate environment of the odorant source is reduced compared to control flies. Flight exploration normally allows insects to sample the quality of potential food sources and to detect predators^56^,^57^. Reaching a food source a handful of seconds faster than control flies is not a decisive advantage since it generally requires at least several minutes to find a mate or to lay eggs on a new food source. Moreover, the faster flight of the mutant was somewhat cancelled by its delayed lift-up latency. Drosophila flight is a sophisticated behavior that could be compared to the complex courtship ritual involving a multiple exchange of sensory informations between the two sex partners^58^. A “too” fast mating process may be maladaptive for the female, which has a limited number of partners, and therefore needs to discriminate the “best” male^50^,^59^.

In summary, our study reveals that free-flight odor tracking can be modulated by wing chemosensors and by *desat1*-expressing tissues. Flight is an exquisite behavior consisting of a series of behavioural sequences all of which depends on a fine genetic control in multiple tissues. Therefore, insect flight results of a high evolutionary adaptation to environmental constraints implying the intricate integration of diverse cues including complex chemosensory signals.

## Methods

### Drosophila stocks

All *Drosophila melanogaster* genotypes were raised on yeast/cornmeal/agar medium and kept at 24 ± 0.5° with 65 ± 5% humidity on a 12 L: 12 D cycle (subjective day 8:00am–8:00 pm). The Dijon2000 (Di2) wild-type strain was collected in Dijon, France in the year 2000.

The WinGal4 strain contains a PGal4 element inserted in the *Serrate* gene (Bloomington #6791) allowing us to express the GAL4 factor only in the larval wing imaginal disc and in the adult sensory organs of the anterior wing margin^27^. Therefore, the WinGal4 transgene was used as a tissue-specific driver to specifically target (in wing sensory neurons) the UAS-reporter transgenes described below. The UAS-*Poxn* RNAi transgene, purchased from the Vienna Drosophila Resource Center (VDRC; #107471) was used to down-regulate the product of the *poxneuro* gene (*poxn*) normally required for sensory organs determination. Defective Poxn expression led to the transformation of chemosensory cells into mechanosensory cells^27^,^60^. UAS-RNAi transgenic for gustatory receptor (GRs; VDRC: GR59f = #101709) was chosen based on the presence of this GR in the anterior wing margin^27^,^61^. The UAS-*tra* (Tra^F^) transgene contains the female-specific isoform of the *transformer* gene which dominantly and cell-autonomously feminizes tissues targeted by GAL4^62^.

We also used *desat1-Gal4* transgenics. The *desat1*-1573-Gal4 mutant allele (“1573”) contains a P-Gal4 transposon inserted in the regulatory region of *desat1*; homozygous 1573/1573 flies are defective both for their production and perception of cuticular sex pheromones^32^,^37^,^38^. The 1573 allele was also used to target (in heterozygous 1573/+ flies) the UAS-*desat1*-RNAi (VDRC; #33338) allowing us to down regulate *desat1* expression. The UAS-*desat1*-RNAi was also targeted by the complete *desat1* regulatory region (6908-Gal4^32^,^38^). Since 6908/RNAi individuals die during larval development, we rescued adulthood by feeding larvae with oleic acid (C18:1; 500 mg/ml) added in the lab food^39^. After emergence, rescued 6908/RNAi adults were either shifted on standard food or kept on C18:1-rich food. All these UAS and Gal4 transgenes were isogenized in the genetic background of the Cs strain prior to the tests to homogenize their genetic background.

### Wind tunnel

The design of wind tunnel has been previously described in detail^63^. Briefly, this is a commercial open circuit, closed throat wind tunnel (Engineering Laboratory Design, Inc., Lake City, MN, USA), equipped with a real-time 3D tracking system. Flight trajectories were measured at 50 Hz with a “Trackit” system (SciTrackS GmbH, Bertschikon, Switzerland) using two cameras (Samsung SHC-735). The wind tunnel contains a laminar airflow with a test section made of clear acrylic (length = 1.55 m; width and height = 0.305 m). The tunnel was illuminated by four bands strip of White LED (BDL-F300W-05-3528, Boulevard des Leds, France; length = 1 m) localized below the active part of the tunnel base (corresponding to the part where free flight was filmed) and separated with a red screen. A tracing paper was used over the tunnel to homogenize the light shining inside the test section. The two lateral panels of the tunnel were covered with a randomized pattern consisting of black and white squares (side = 3 cm). In the upwind section of active section, a platform (height = 5 cm; ø = 4 cm) was placed exactly halfway between the tunnel sides. Odorants were deposited on the platform, before each experiment. Wind velocity was set to 0.4 m.s^−1^.

### Odors

In the first series of experiments, Cs flies were tested towards several odorant molecules to determine their attractiveness (Fig. 1). Acetic acid and methylene chloride (CH_2_Cl_2_) were purchased from Sigma-Aldrich (St. Louis, MO, USA), and hydroxy-3-butanone-2 (acetoin) from Cayman chemical company (Michigan, USA). We also used pure grape juice (Eco+, Leclerc, France) and dry baker yeast (Briochin, Alsa, France). For each test, 250 μl or 5 g of tested odorant were deposited on a thin glass slide (2 × 2 cm), which was immediately placed on top of the platform. For “plain food” experiments, small Whatman filter paper patches (length = 3.5 cm; width = 1.5 cm; GE healthcare Life sciences) were dipped into freshly prepared plain food during the 24 h period preceding the test; the excess of food was removed with a spatula, just prior to the test. For “acetoin” experiments, plain food patches were impregnated (few minutes before each experiment), either with 80 μl of CH_2_Cl_2_ alone (solvent) or mixed with acetoin (400ng or 800 μg). For all genotypes, we used water as a control substance, and plain food as an experimental attractive stimulus. For the sake of clarity, we show most “water experiments” in the Supplementary section.

### Behavior

All tester flies were isolated 0–4 h after eclosion under light CO_2_ anaesthesia. Male and females were held individually in fresh glass food vials during 3 to 7days prior to testing. 20–24 h before the test, flies were food-deprived (and kept in a humidified chamber) in order to motivate flight. Experiments were always performed at 24 ± 0.5 °C with 65 ± 5% humidity, from 8am to noon. Tester flies were individually aspirated (without anaesthesia) and introduced inside a vial connected to an acrylic tube (ø = 5 mm), separated by a gate, for 3 min acclimation. Then, each tester fly was allowed to walk inside this tube (approximately located at half height of the tunnel) to reach the “active” section of the wind tunnel. Each experiment lasted 10 min or less when the fly landed on the platform.

To characterize free-flight toward potentially attractive odors, we successively measured the lift-up frequency and latency, the period of time spent in the “active” section of the tunnel, the landing frequency (of flying insects), and the total duration of flight (time lapse between lift-up and landing). The acquisition of 3-dimensional (3-D) flight trajectory was calculated in real-time based on a published algorithm^64^. Trajectories were further processed, filtered and analyzed using MATLAB or OCTAVE softwares. Flight trajectories were described in terms of a number of variables such as the distance travelled, velocity, heading and angular velocity, which were calculated on every frame for all trajectories^18^. Transit probability histograms were used to visualize the distribution of flies relatively to each tunnel axis (X, Y, Z; Fig. 4a,b). For each condition, all individual trajectories were overloaded and derived by dividing two-dimensional views into squares with 1 cm-side length to produce cross-over representation (Fig. 1c). More precisely, the number of fly occurrences within each square (cross-over) was summed and divided by the total number of fly occurrences in all squares, this yielding the probability of square occupancy. To enhance such visualization, the threshold of transit probability was set up at 0.4% for the maximum. To prevent outlier values and for further analysis, we only kept 80% of trajectories. Our characterization was based on 12 to 41 trajectories.

### Statistics

All statistical analyses were performed using XLSTAT 2012 software (Addinsoft, XLSTAT 2012) and customs scripts on MATLAB. Data points obtained for the percentages of lift-up and of landing in active flies were compared with a Fisher exact test and are shown as histogram bars. The inter-genotype comparison for parameters such as the lift-up latency, flight duration and distance traveled was assayed with a Kruskall-Wallis test and their variability represented with box-whisker plots. The *post-hoc* test were performed with the Steel-Dwass-Critchlow-Fligner procedure with a Bonferroni correction The velocity, heading flight and angular velocity parameters were compared using N-way analyses of variance (ANOVA_n_) completed by a multiple pairwise comparisons using Fisher *post-hoc* tests. Only *P* values < 0.05 were considered to be statistically significant.

## Additional Information

**How to cite this article**: Houot, B. *et al*. Free flight odor tracking in Drosophila: Effect of wing chemosensors, sex and pheromonal gene regulation. *Sci. Rep.* **7**, 40221; doi: 10.1038/srep40221 (2017).

**Publisher's note:** Springer Nature remains neutral with regard to jurisdictional claims in published maps and institutional affiliations.

## Supplementary Material

Supplementary Information
