# Supplementary material for: Free flight odor tracking in Drosophila: Effect of wing chemosensors, sex and pheromonal gene regulation
Source: Sci Rep. 2017 Jan 9;7:40221. doi: 10.1038/srep40221 (PMC5220339; doi:10.1038/srep40221)
Supplement: Supplementary Information [file srep40221-s1.pdf]

Supplementary Information for:

Free flight odor tracking in Drosophila: Effect of wing chemosensors, sex and pheromonal gene regulation

By: Benjamin Houot[1], Vincent Gigot[1], Alain Robichon[2], Jean-François Ferveur[1*]

Supplementary Figures

Supplementary Figure S1**. Free-flight of transgenic Drosophila flies with genetically altered wing margin chemosensors to water.** Histograms and box-whisker plots represents four free-flight features to water. See also the complete statistical analysis on Supplementary Table S2. For more information, please refer to the legend of Figs.1 and 2. N=21-41. The same genotypes were tested with plain food (Fig. 2).

Supplementary Figure S2. **Free-flight to water in *desat1* transgenic Drosophila flies.** Histograms and box-whisker plots represent four free-flight features to water. See the complete statistical analysis on Supplementary Table S4. The same genotypes were tested with plain food (Fig. 3). For more information, please refer to the legends of Figs. 1 and 3. N=35-65.

Supplementary Figure S3. **Spatial distribution of control and mutant flies during free-flight to water.** (**a**) Histograms represent the distribution of flies during flight according to each axis of the tunnel: X=length, Y=width, Z=heigh (Cs control

flies=blue color; 1573 mutants=yellow; their overlap=grey), (**b**) For each sex (male=left; female=right) and genotype (Cs control flies on top; 1573 mutant at the bottom), we provide both XY (top) and XZ (bottom) two-dimensional representation of the probability to find a fly in the tunnel. N= 12-20. (The response to plain food of these genotypes is shown in Fig. 3).

Supplementary Figure S4**. Additional free-flight parameters to compare control and mutant *desat1*-1573 flies.** Histograms represent the mean (±s.e.m.) of four additional free-flight features measured in control (Cs, left bars) and mutant (1573/1573) flies tested to water and plain food. (**a-c**) Male and female flies are shown as empty and shaded bars, respectively. (**a**) represents the distance (in meters) made by flies between lifting-up and landing, (**b**) is the velocity (in meter/sec), (**c**) is the heading flight (measured in degrees). (**d**) For the measure of angular velocity (degrees/sec), sexes and food types, which showed no difference, were pooled. (**a-c**) For each parameter, significant intra-sex differences were determined with a Kruskall-Wallis test (for distance traveled) and with an ANOVA with multiple pairwise comparaisons followed by Fisher post-hoc tests (for velocity, heading flight and angular velocity). Differences are indicated by different letters (lower cases for males; capital letters for females). Inter-sex differences were determined with a χ^2homogeneity test (*$p<0.05$). N=12-41.

Supplementary Figure S5. **Polar histograms representing the heading directionality in wild-type and 1573 mutant flies.** Polar histograms represent

free-flight directionality in male (**a**) and female (**b**) flies of control (Cs) and *desat1* mutant (1573) strains. The distribution of heading angles (shown by numbers around each circle) indicate directionality. The upwind direction correspond to a imaginary line along the tunnel X axis from the "-180" to "0" angle. Flies were either tested in the presence of water (blue color) or plain food (magenta). N= 12-28.

Supplementary Tables

Supplementary Table S1. **Statistical level of significance (Kruskal Wallis test) for (a) « Lift up Latency » and (b) «Flight duration » to Plain food (Corresponding data are shown on Figure 2).** The statistical comparison was carried out between males (m) and females (F) of three genotypes groups (« Poxn », « Gr59f », « Tra^{F} »). Each comparison involved the common control genotype WinGal4. For more details on genotypes, please refer to the Figure 2 and to the Material and methods section. Statistically significant differences are shown in bold characters.

Supplementary Table S2. **Statistical level of significance (Kruskal Wallis test) for « Lift up Latency » to water (Corresponding data are shown on Supplementary Fig.S1).** For more information, please refer to the legend of Supplementary Table S1.

Supplementary Table S3. **Statistical level of significance (Kruskal Wallis test) for (a) « Lift up Latency » and (b) «Flight duration » to Plain food (Corresponding**

data are shown on Figure 3). For more information, please refer to the legend of Figure 3 and Supplementary Table S1 and to the Material and methods section.

Supplementary Table S4. **Statistical level of significance (Kruskal Wallis test) for « Lift up Latency » to water (Corresponding data are shown on Supplementary Fig.S2).** For more information, please refer to the legend of Supplementary Table S1.

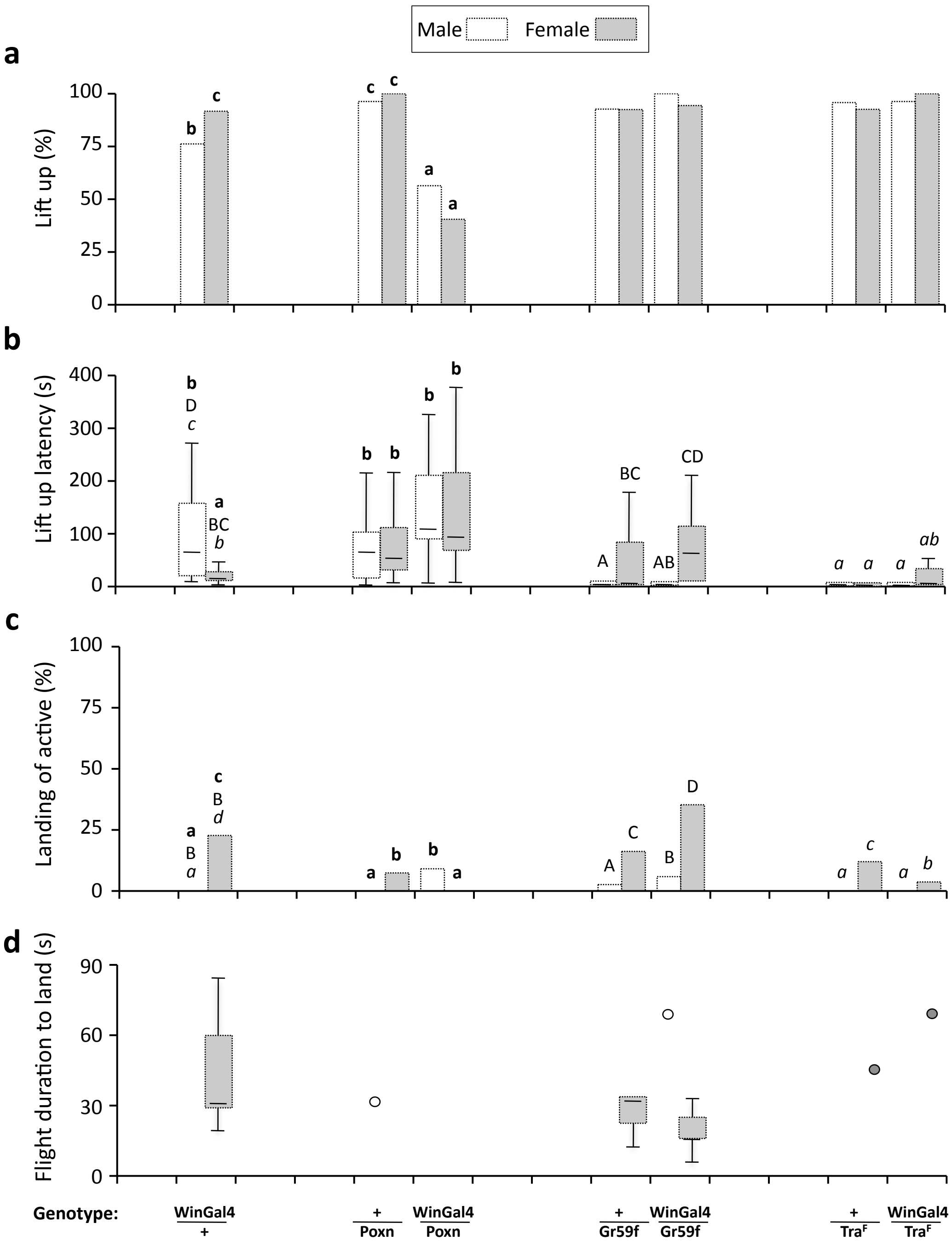

Supplemental Figure 1

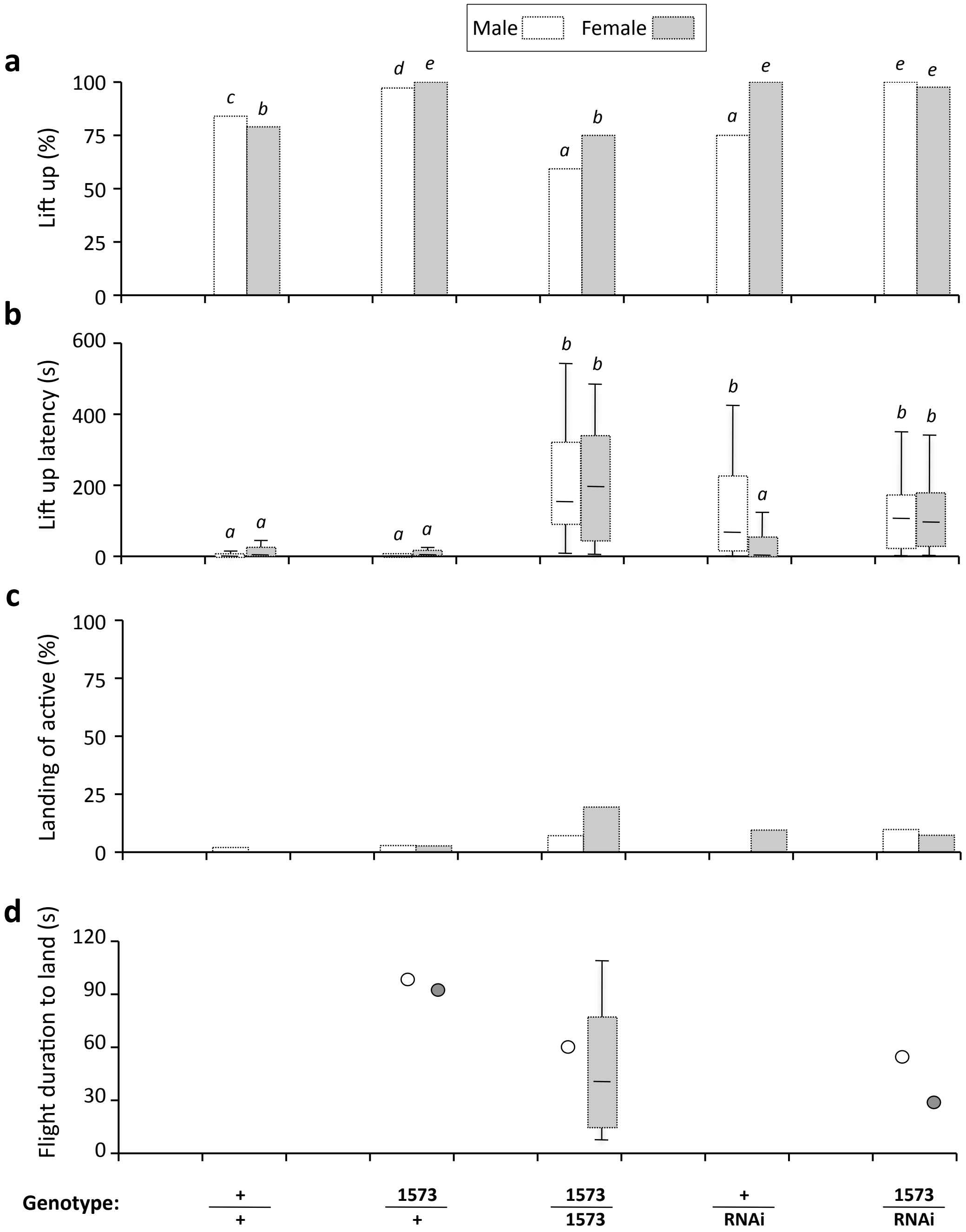

Supplemental Figure 2

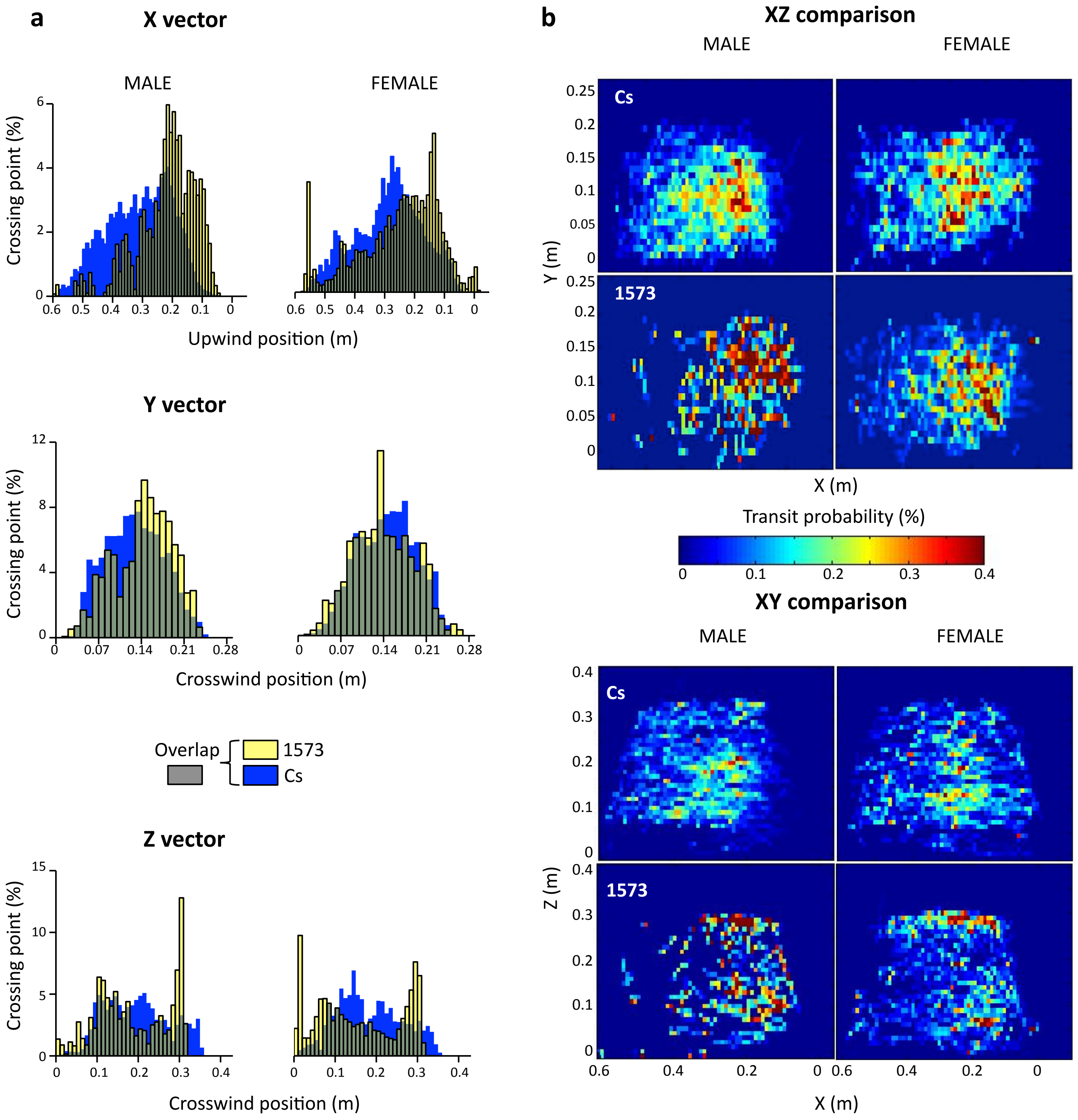

Supplemental Figure 3

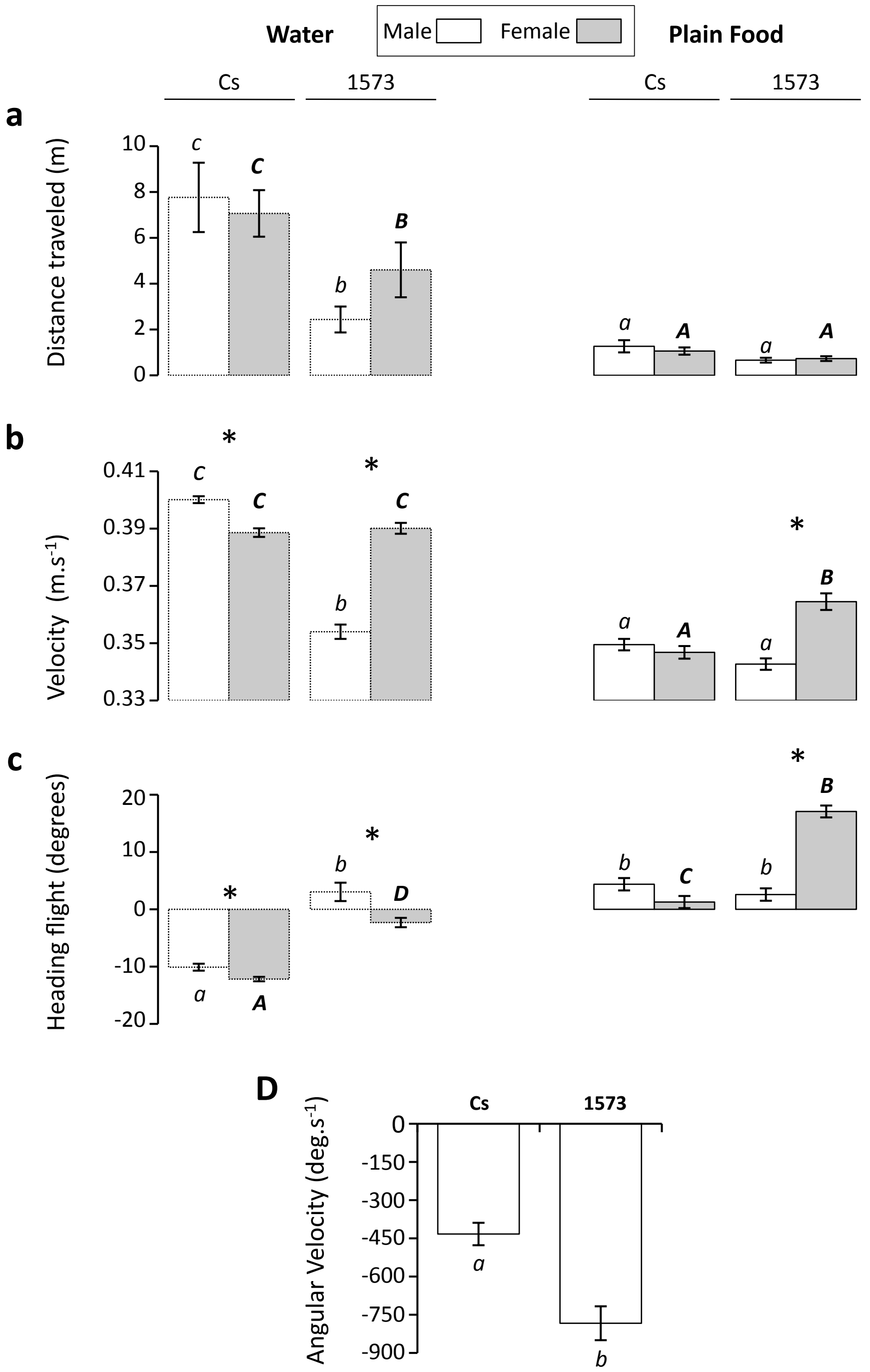

Supplemental Figure 4

a Male

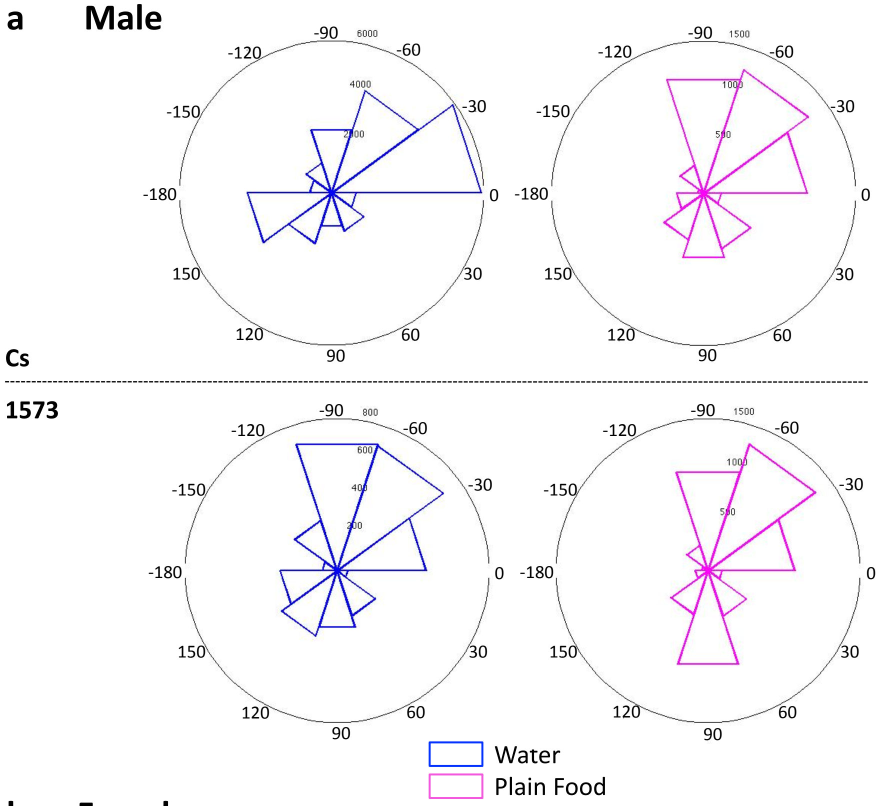

b Female

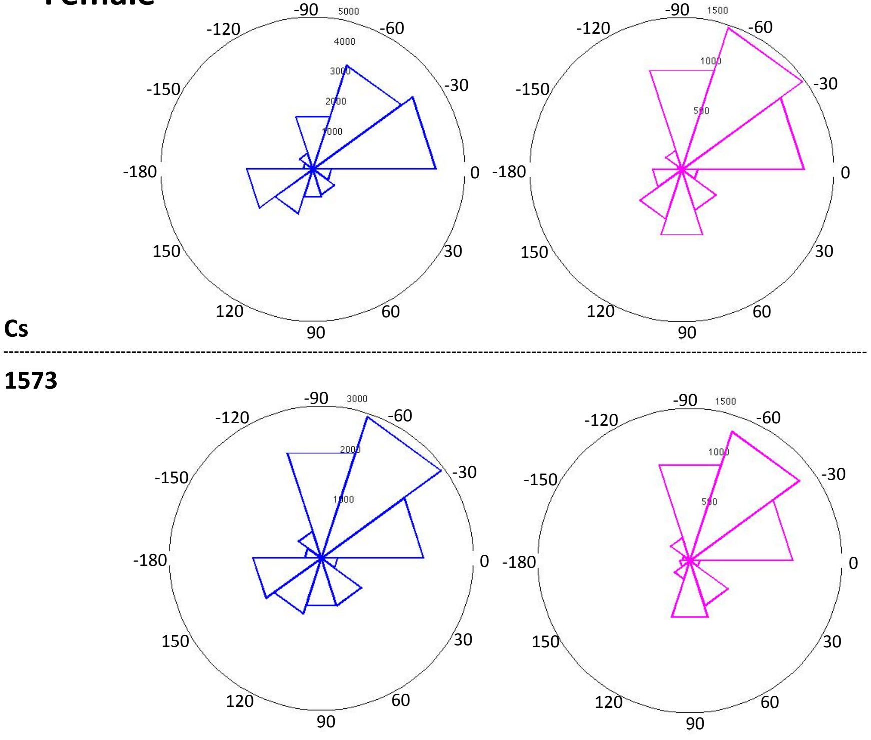

Table S1a. Statistical level of significance (Kruskal Wallis test) for "Lift up Latency" to Plain food (data shown on Figure 2).

	m WinGal4/+	f WinGal4/+	m +/Poxn	f +/Poxn	m WinGal4/Poxn	f WinGal4/Poxn
m WinGal4/+	1	**0.026**	0.732	0.283	1.000	0.992
f WinGal4/+	**0.026**	1	0.255	0.944	**0.009**	**0.006**
m +/Poxn	0.732	0.255	1	0.933	0.676	0.621
f +/Poxn	0.283	0.944	0.933	1	0.171	0.093
m WinGal4/Poxn	1.000	**0.009**	0.676	0.171	1	0.996
f WinGal4/Poxn	0.992	**0.006**	0.621	0.093	0.996	1

	m WinGal4/+	f WinGal4/+	m +/Gr59f	f +/Gr59f	m WinGal4/Gr59f	f WinGal4/Gr59f
m WinGal4/+	1	**0.026**	**0.028**	**< 0.0001**	0.075	0.246
f WinGal4/+	**0.026**	1	0.925	0.165	0.999	0.971
m +/Gr59f	**0.028**	0.925	1	0.536	0.997	0.118
f +/Gr59f	**< 0.0001**	0.165	0.536	1	0.294	**0.002**
m WinGal4/Gr59f	0.075	0.999	0.997	0.294	1	0.539
f WinGal4/Gr59f	0.246	0.971	0.118	**0.002**	0.539	1

	m WinGal4/+	f WinGal4/+	m +/Tra^f	f +/Tra^f	m WinGal4/Tra^f	f WinGal4/Tra^f
m WinGal4/+	1	**0.026**	**0.000**	**< 0.0001**	0.454	0.112
f WinGal4/+	**0.026**	1	0.373	**0.045**	0.860	0.997
m +/Tra^f	**0.000**	0.373	1	0.964	**0.014**	0.063
f +/Tra^f	**< 0.0001**	**0.045**	0.964	1	**0.001**	**0.005**
m WinGal4/Tra^f	0.454	0.860	**0.014**	**0.001**	1	0.974
f WinGal4/Tra^f	0.112	0.997	0.063	**0.005**	0.974	1

Table S1b. Statistical level of significance (Kruskal Wallis test) for “Flight duration” to Plain food (data shown on Figure 2).

	m WinGal4/+	f WinGal4/+	m +/Poxn	f +/Poxn	m WinGal4/Poxn	f WinGal4/Poxn
m WinGal4/+	1	**0.001**	0.297	0.371	0.661	**0.028**
f WinGal4/+	**0.001**	1	0.403	0.293	**0.039**	0.850
m +/Poxn	0.297	0.403	1	1.000	0.930	0.993
f +/Poxn	0.371	0.293	1.000	1	0.980	0.931
m WinGal4/Poxn	0.661	**0.039**	0.930	0.980	1	0.462
f WinGal4/Poxn	**0.028**	0.850	0.993	0.931	0.462	1

	m WinGal4/+	f WinGal4/+	m +/Gr59f	f +/Gr59f	m WinGal4/Gr59f	f WinGal4/Gr59f
m WinGal4/+	1	**0.001**	0.987	0.180	0.830	**0.000**
f WinGal4/+	**0.001**	1	**0.002**	0.366	**< 0.0001**	0.998
m +/Gr59f	0.987	**0.002**	1	0.238	0.243	**0.000**
f +/Gr59f	0.180	0.366	0.238	1	**0.001**	0.072
m WinGal4/Gr59f	0.830	**< 0.0001**	0.243	**0.001**	1	**< 0.0001**
f WinGal4/Gr59f	**0.000**	0.998	**0.000**	0.072	**< 0.0001**	1

	m WinGal4/+	f WinGal4/+	m +/Tra^f	f +/Tra^f	m WinGal4/Tra^f	f WinGal4/Tra^f
m WinGal4/+	1	**0.001**	0.998	0.994	1.000	1.000
f WinGal4/+	**0.001**	1	**< 0.0001**	**< 0.0001**	**< 0.0001**	**0.001**
m +/Tra^f	0.998	**< 0.0001**	1	0.999	1.000	0.998
f +/Tra^f	0.994	**< 0.0001**	0.999	1	0.999	0.985
m WinGal4/Tra^f	1.000	**< 0.0001**	1.000	0.999	1	1.000
f WinGal4/Tra^f	1.000	**0.001**	0.998	0.985	1.000	1

Table S2. Statistical level of significance (Kruskal Wallis test) for “Lift up Latency” to Water (data shown on Supplemental Figure S1)

	m WinGal4/+	f WinGal4/+	m +/Poxn	f +/Poxn	m WinGal4/Poxn	f WinGal4/Poxn
m WinGal4/+	1	**0.035**	0.998	0.998	0.669	0.772
f WinGal4/+	**0.035**	1	0.191	**0.027**	**0.001**	**0.002**
m +/Poxn	0.998	0.191	1	1.000	0.344	0.609
f +/Poxn	0.998	**0.027**	1.000	1	0.096	0.337
m WinGal4/Poxn	0.669	**0.001**	0.344	0.096	1	0.997
f WinGal4/Poxn	0.772	**0.002**	0.609	0.337	0.997	1

	m WinGal4/+	f WinGal4/+	m +/Gr59f	f +/Gr59f	m WinGal4/Gr59f	f WinGal4/Gr59f
m WinGal4/+	1	**0.035**	**< 0,0001**	**0.041**	**0.002**	0.974
f WinGal4/+	**0.035**	1	**0.000**	0.742	0.061	0.393
m +/Gr59f	**< 0,0001**	**0.000**	1	**0.012**	0.680	**< 0,0001**
f +/Gr59f	**0.041**	0.742	**0.012**	1	0.447	0.230
m WinGal4/Gr59f	**0.002**	0.061	0.680	0.447	1	**0.004**
f WinGal4/Gr59f	0.974	0.393	**< 0,0001**	0.230	**0.004**	1

	m WinGal4/+	f WinGal4/+	m +/Tra^f	f +/Tra^f	m WinGal4/Tra^f	f WinGal4/Tra^f
m WinGal4/+	1	**0.035**	**0.000**	**< 0,0001**	**< 0,0001**	**0.004**
f WinGal4/+	**0.035**	1	**0.004**	**0.006**	**0.001**	0.709
m +/Tra^f	**0.000**	**0.004**	1	1.000	0.849	0.172
f +/Tra^f	**< 0,0001**	**0.006**	1.000	1	0.918	0.209
m WinGal4/Tra^f	**< 0,0001**	**0.001**	0.849	0.918	1	0.369
f WinGal4/Tra^f	**0.004**	**0.709**	0.172	0.209	0.369	1

Table S3a. Statistical level of significance (Kruskal Wallis test) for “Lift up Latency” to Plain food (data shown on Figure 3).

).	m +/+	f +/+	m 1573/1573	f 1573/1573	m 1573/+	f 1573/+	m +/RNAi	f +/RNAi	m 1573/RNAi	f 1573/RNAi	f L_6908/RNAi	m L_6908/RNAi	f L+A_6908/RNAi	m L+A_6908/RNAi
m +/+	1	1.000	**0.000**	**0.000**	**0.001**	0.307	0.179	0.956	**< 0.0001**	**< 0.0001**	**< 0.0001**	**< 0.0001**	**0.000**	**0.003**
f +/+	1.000	1	**0.000**	**< 0.0001**	**0.001**	0.335	0.343	0.969	**< 0.0001**	**< 0.0001**	**< 0.0001**	**< 0.0001**	**0.000**	**0.006**
m 1573/1573	**0.000**	**0.000**	1	1.000	**< 0.0001**	**< 0.0001**	0.939	**0.000**	0.999	1.000	1.000	0.998	1.000	0.933
f 1573/1573	**0.000**	**< 0.0001**	1.000	1	**< 0.0001**	**< 0.0001**	0.861	**0.000**	0.999	1.000	0.999	0.984	1.000	0.886
m 1573/+	**0.001**	**0.001**	**< 0.0001**	**< 0.0001**	1	0.994	**< 0.0001**	0.339	**< 0.0001**	**< 0.0001**	**< 0.0001**	**< 0.0001**	**< 0.0001**	**< 0.0001**
f 1573/+	0.307	0.335	**< 0.0001**	**< 0.0001**	0.994	1	**0.001**	0.992	**< 0.0001**	**< 0.0001**	**< 0.0001**	**< 0.0001**	**< 0.0001**	**< 0.0001**
m +/RNAi	0.179	0.343	0.939	0.861	**< 0.0001**	**0.001**	1	**0.021**	0.463	0.752	0.265	0.294	0.916	1.000
f +/RNAi	0.956	0.969	**0.000**	**0.000**	0.339	0.992	**0.021**	1	**< 0.0001**	**< 0.0001**	**< 0.0001**	**< 0.0001**	**0.001**	**0.003**
m 1573/RNAi	**< 0.0001**	**< 0.0001**	0.999	0.999	**< 0.0001**	**< 0.0001**	0.463	**< 0.0001**	1	0.997	1.000	1.000	0.896	0.351
f 1573/RNAi	**< 0.0001**	**< 0.0001**	1.000	1.000	**< 0.0001**	**< 0.0001**	0.752	**< 0.0001**	0.997	1	0.997	0.972	1.000	0.969
f L_6908/RNAi	**< 0.0001**	**< 0.0001**	1.000	0.999	**< 0.0001**	**< 0.0001**	0.265	**< 0.0001**	1.000	0.997	1	1.000	0.871	0.170
m L_6908/RNAi	**< 0.0001**	**< 0.0001**	0.998	0.984	**< 0.0001**	**< 0.0001**	0.294	**< 0.0001**	1.000	0.972	1.000	1	0.645	0.146
f L+A_6908/RNAi	**0.000**	**0.000**	1.000	1.000	**< 0.0001**	**< 0.0001**	0.916	**0.001**	0.896	1.000	0.871	0.645	1	0.997
m L+A_6908/RNAi	**0.003**	**0.006**	0.933	0.886	**< 0.0001**	**< 0.0001**	1.000	**0.003**	0.351	0.969	0.170	0.146	0.997	1

Table S3b. Statistical level of significance (Kruskal Wallis test) for “Flight duration” to Plain food (data shown on Figure 3).

	m +/+	f +/+	m 1573/1573	f 1573/1573	m 1573/+	f 1573/+	m +/RNAi	f +/RNAi	m 1573/RNAi	f 1573/RNAi	f L_6908/RNAi	m L_6908/RNAi	f L+A_6908/RNAi	m L+A_6908/RNAi
m +/+	1	0.999	0.762	**0.039**	**0.000**	**< 0.0001**	1.000	0.836	**< 0.0001**	**< 0.0001**	0.328	**0.011**	0.550	0.078
f +/+	0.999	1	0.951	0.066	**< 0.0001**	**< 0.0001**	0.776	0.967	**< 0.0001**	**< 0.0001**	0.215	**0.005**	0.742	**0.045**
m 1573/1573	0.762	0.951	1	1.000	0.562	**0.005**	0.618	1.000	0.243	**0.013**	1.000	1.000	1.000	1.000
f 1573/1573	**0.039**	0.066	1.000	1	0.375	**0.000**	**0.008**	0.986	**0.049**	**0.000**	1.000	1.000	0.992	1.000
m 1573/+	**0.000**	**< 0.0001**	0.562	0.375	1	0.385	**< 0.0001**	**0.043**	1.000	0.928	0.136	0.635	**0.028**	0.589
f 1573/+	**< 0.0001**	**< 0.0001**	**0.005**	**0.000**	0.385	1	**< 0.0001**	**< 0.0001**	0.621	0.993	**< 0.0001**	**0.000**	**< 0.0001**	**0.000**
m +/RNAi	1.000	0.776	0.618	**0.008**	**< 0.0001**	**< 0.0001**	1	0.435	**< 0.0001**	**< 0.0001**	**0.034**	**0.000**	0.066	**0.006**
f +/RNAi	0.836	0.967	1.000	0.986	**0.043**	**< 0.0001**	0.435	1	**0.002**	**< 0.0001**	1.000	0.939	1.000	0.980
m 1573/RNAi	**< 0.0001**	**< 0.0001**	0.243	**0.049**	1.000	0.621	**< 0.0001**	**0.002**	1	0.982	**0.002**	0.056	**0.000**	0.101
f 1573/RNAi	**< 0.0001**	**< 0.0001**	**0.013**	**0.000**	0.928	0.993	**< 0.0001**	**< 0.0001**	0.982	1	**< 0.0001**	**0.001**	**< 0.0001**	**0.001**
f L_6908/RNAi	0.328	0.215	1.000	1.000	0.136	**< 0.0001**	**0.034**	1.000	**0.002**	**< 0.0001**	1	1.000	1.000	0.998
m L_6908/RNAi	**0.011**	**0.005**	1.000	1.000	0.635	**0.000**	**0.000**	0.939	0.056	**0.001**	1.000	1	0.932	1.000
f L+A_6908/RNAi	0.550	0.742	1.000	0.992	**0.028**	**< 0.0001**	0.066	1.000	**0.000**	**< 0.0001**	1.000	0.932	1	0.946
m L+A_6908/RNAi	0.078	**0.045**	1.000	1.000	0.589	**0.000**	**0.006**	0.980	0.101	**0.001**	0.998	1.000	0.946	1

Table S4. Statistical level of significance (Kruskal Wallis test) for “Lift up Latency” to water (data shown on Supplemental Figure 2)

	m +/+	f +/+	m 1573/1573	f 1573/1573	m 1573/+	f 1573/+	m +/RNAi	f +/RNAi	m 1573/RNAi	f 1573/RNAi
m +/+	1	0.300	**< 0.0001**	**< 0.0001**	1.000	0.809	**< 0.0001**	0.293	**< 0.0001**	**< 0.0001**
f +/+	0.300	1	**< 0.0001**	**< 0.0001**	0.545	0.995	**0.003**	1.000	**0.000**	**< 0.0001**
m 1573/1573	**< 0.0001**	**< 0.0001**	1	1.000	**< 0.0001**	**< 0.0001**	0.391	**< 0.0001**	0.356	0.514
f 1573/1573	**< 0.0001**	**< 0.0001**	1.000	1	**< 0.0001**	**< 0.0001**	0.594	**< 0.0001**	0.411	0.724
m 1573/+	1.000	0.545	**< 0.0001**	**< 0.0001**	1	0.968	**< 0.0001**	0.613	**< 0.0001**	**< 0.0001**
f 1573/+	0.809	0.995	**< 0.0001**	**< 0.0001**	0.968	1	**0.000**	0.998	**0.000**	**< 0.0001**
m +/RNAi	**< 0.0001**	**0.003**	0.391	0.594	**< 0.0001**	**0.000**	1	**0.006**	1.000	1.000
f +/RNAi	0.293	1.000	**< 0.0001**	**< 0.0001**	0.613	0.998	**0.006**	1	**0.002**	**0.000**
m 1573/RNAi	**< 0.0001**	**0.000**	0.356	0.411	**< 0.0001**	**0.000**	1.000	**0.002**	1	1.000
f 1573/RNAi	**< 0.0001**	**< 0.0001**	0.514	0.724	**< 0.0001**	**< 0.0001**	1.000	**0.000**	1.000	1
